# Autophagy in Plasma Cell Ontogeny and Malignancy

**DOI:** 10.1007/s10875-016-0254-9

**Published:** 2016-03-16

**Authors:** Enrico Milan, Monica Fabbri, Simone Cenci

**Affiliations:** Division of Genetics and Cell Biology, San Raffaele Scientific Institute, Milano, Italy; Università Vita-Salute San Raffaele, Milano, Italy

**Keywords:** Antibody, autophagy, blimp-1, endoplasmic reticulum, immunoglobulin, multiple myeloma, p62, plasma cell, proteasome, SQSTM1, ubiquitin

## Abstract

Autophagy is a highly conserved pathway that recycles cytosolic material and organelles via lysosomal degradation. Once simplistically viewed as a non-selective survival strategy in dire straits, autophagy has emerged as a tightly regulated process ensuring organelle function, proteome plasticity, cell differentiation and tissue homeostasis, with key roles in physiology and disease. Selective target recognition, mediated by specific adapter proteins, enables autophagy to orchestrate highly specialized functions in innate and adaptive immunity. Among them, the shaping of plasma cells for sustainable antibody production through a negative control on their differentiation program. Moreover, memory B cells and long-lived plasma cells require autophagy to exist. Further, the plasma cell malignancy, multiple myeloma deploys abundant autophagy, essential for homeostasis, survival and drug resistance.

## Introduction

Plasma cells (PCs) are terminal immune effectors of the B lymphocyte lineage devoted to massive immunoglobulin (Ig) synthesis and secretion. Their differentiation entails profound genetic reprogramming and cellular reshaping, and imposes intense stress, counterbalanced by *ad hoc* adaptive strategies. Macroautophagy (conventionally referred to as *autophagy*) is a conserved intracellular membrane trafficking process that engulfs unwanted supra-molecular entities and directs them to lysosomes for degradation and recycling. In addition to its prime metabolic role maintaining energy homeostasis, autophagy evolved diverse complex functions, including proteome and organelle quality control, cell differentiation and stress responses. Recently, a variety of regulatory functions across innate and adaptive immunity have been recognized, including intracellular microbe clearance, inflammation, lymphocyte development and antigen presentation. We disclosed a previously unrecognized role moderating PC differentiation and function, whereby autophagy sustains both short- and long-term humoral immunity. Moreover, multiple myeloma cells proved exquisitely dependent on autophagy. This essay recapitulates and discusses these findings and their pathophysiologic and therapeutic implications in the context of immunity and cancer.

## Autophagy and Autophagic Receptors

Autophagy is a highly conserved intracellular self-digestive pathway that consists in the sequestration of substrates in short-lived double-membrane vesicles, called *autophagosomes*, subsequently delivered to the lysosome (in animal cells) or the vacuole (in yeast and plant cells) for content degradation and recycling. The prime conserved function of autophagy is to sustain cellular metabolism in conditions of nutrient starvation. However, crucial roles have emerged in a wide variety of biological processes such as cell differentiation and death, immunity, and aging. Indeed, the notion of autophagy as a non-selective bulk cytoplasmic degradation pathway triggered by starvation has been challenged and revisited in view of its established capacity to ensure selective elimination of harmful aggregates (*aggrephagy*) and microorganisms (*xenophagy*), as well as homeostatic organelle renewal through dismantlement of mitochondria (*mitophagy*), ribosomes (*ribophagy*), endoplasmic reticulum (*ER-phagy*), peroxysomes (*pexophagy*), and lipid droplets (*lipophagy*) (reviewed in [1]).

Autophagic selectivity is conferred by dedicated receptor/adapter proteins that recognize the substrate and mediate its engulfment into autophagosomes. Autophagy receptors share three key moieties: (i) a ubiquitin (Ub)-binding domain for target recognition; (ii) an LC3-interacting region cross-linking the substrate with the autophagic machinery; (iii) a PB1 domain for polymerization [2]. The degradation signal, ubiquitination, is shared with the Ub proteasome system (UPS); however, while K48-linked poly-Ub chains are recognized by proteasomes, K63-linked Ub chains are preferentially targeted by autophagy [3].

The prototypical autophagic receptor, SQSTM1/p62, has been implicated in many types of selective autophagy. Other receptors include NBR1, optineurin, NDP52 and TAX1BP1 [2]. In addition to the domains required for autophagic activity, p62 harbors multiple signaling moieties, including a ZZ-type zinc finger domain, two nuclear localization signals (NLS), a nuclear export signal (NES), and a KEAP1 interacting region (KIR) [2]. As a result, p62 contributes to shape a number of signaling pathways, including Nrf2, NF-kB, and mTOR [4]. Nrf2 mediates a critical cellular defense against oxidative damage. P62 promotes Nrf2 activity via its inhibitory interaction with KEAP1, a redox-sensitive substrate adapter for a Ub-ligase complex. In unstressed conditions, KEAP1 drives constant ubiquitination and proteasomal degradation of Nrf2. Under stresses that result in accumulation of p62, its increased binding with KEAP1 liberates and activates Nrf2 [5, 6], linking protein and redox homeostasis. P62 has also been shown to modulate programmed cell death. For instance, p62 participates in the full activation of caspase 8 during ligand-induced apoptosis [7]. Thus, p62 is a hub signaling adapter integrating diverse stimuli to coordinate protein homeostasis with different cell and tissue functions. Glucose, lipid and skeletal metabolism depend on p62 [5, 6, 8, 9], as mice lacking p62 display mature-onset insulin resistance and obesity [8]. In neurodegenerative disorders, p62 bound to Ub-proteins is commonly found in diagnostic inclusion bodies, such as Lewy bodies, huntingtin aggregates and neurofibrillary tangles, respectively in Parkinson’s, Huntington’s and Alzheimer’s diseases [10]. P62 mutations, generally mapping to the Ub-binding domain, are associated with altered NF-κB mediated osteoclastogenesis in Paget’s disease of bone, an age-onset chronic skeletal disorder characterized by focal enhanced bone turnover [11]. In line with the complex integrative functions of p62 alluded to above, it is not surprising that, although highly conserved in metazoans, p62 is absent in plants and fungi [1].

## Immune Functions of Autophagy

Autophagy orchestrates many innate and adaptive immune functions, including elimination of microorganism, control of inflammation, secretion of immune mediators, antigen presentation, and lymphocyte development [12, 13].

Clearance of invading microbes through autophagy (*xenophagy*, *i.e.* eating the stranger) appears the most primordial immune response against intracellular pathogens. Invading microorganisms trigger autophagy via starvation induced by nutrient competition, or through receptors such as toll-like receptors [14]. Infected cells then activate LC3-associated phagocytosis (LAP), which in turn drives phagosome-lysosome fusion and subsequent degradation of invading bacteria [15]. Autophagy receptors can initiate xenophagy by recognizing specific modifications of cytosolic bacteria, such as ubiquitination, binding to galectin, or pathogen-associated lipid changes [1, 16]. Many mechanisms evolved to circumvent eukaryotic control, witnessing the relevance of autophagy against bacteria [12]. Autophagy also mediates viral recognition and destruction. For example, capsid proteins of the neurotropic Sindbis virus are degraded via p62-dependent autophagy [17]. Thus, SQSTM1/p62-like receptors (SLRs) have been proposed to constitute a new family of innate pattern recognition receptors [12, 13].

Autophagy also regulates the inflammatory response by modulating the activity of inflammasomes, cytosolic signaling complexes that promote proteolytic processing and secretion of the pro-inflammatory cytokines IL-1β and IL-18 [12]. These are leaderless proteins secreted through a non-conventional and not fully elucidated process requiring the autophagic machinery [18]. The autophagic control of inflammation is variegated. While in basal conditions autophagy prevents inflammation by limiting mitochondrial production of reactive oxygen species (ROS) and clearing pro-inflammatory protein aggregates [19], upon exposure to damage- or pathogen-associated molecular patterns (DAMPs or PAMPs), autophagy mediates secretion of IL-1β, IL-18 and HMGB1, critical for the prompt establishment of a multicellular inflammatory response [18]. At the same time, autophagy limits excess inflammation by degrading inflammasomes and pro-IL-1β [20].

Autophagy plays important functions also in adaptive immunity. One exemplar role is the regulation of lymphocyte ontogenesis. Critical to T lymphocyte homeostasis, autophagy sustains T cell survival upon TCR activation, and participates in the selection of the T cell repertoire and in T cell maturation [21, 22]. Autophagic clearance of damaged mitochondria is essential in hematopoietic stem cells and for post-thymic T cell maturation [22, 23]. Indeed, the maintenance of normal numbers of CD4+ and CD8+ T cells requires functional Atg proteins [24]. In activated T cells, autophagy sustains ATP levels, controls proliferation and cytokine release [25]. Autophagy may also be involved in Th polarization, as suggested by a model of *M. tubercolosis* infection, where lung autophagy-deficient myeloid cells secreted higher amounts of IL-17 [24, 26].

In the B lymphocyte lineage, autophagy influences transition of pro- to pre-B cells. Moreover, mice lacking the essential autophagy gene Atg5 in mature B cells show fewer B-1a cells in the periphery [27, 28].

A large number of studies have linked autophagy to MHC class I and class II antigen presentation [29–31]. In particular, autophagy increases presentation and citrullination of exogenous viral components and cytoplasmic self-antigens, contributing to the elimination of self-reactive T cells during thymic maturation. Moreover, LAP directs exogenous antigens into the antigen processing compartment [21]. Autophagy also mediates cross-presentation of phagocytosed antigens on MHC class I to prime CD8^+^ T cells *in vivo* [29–31], and may influence MHC class I presentation by competing with the proteasome for substrates [32]. However, autophagy is not a universal antigen-presenting pathway, being, for example, dispensable for presentation by B cells to cognate T cells in the germinal center [28].

## Autophagy in Plasma Cell Ontogeny

PCs, terminal effectors of the B lymphocyte lineage specialized in large-scale antibody secretion, constitute the humoral arm of adaptive immunity. Upon antigen encounter, B cells get activated and start a complex program in secondary lymphoid organs culminating in PC differentiation. Most antibody secreting cells (ASCs) are short-lived and die within few days, providing immediate defense against invading microorganisms [33]. During T cell-dependent immune responses, activated follicular B cells in spleen and lymph nodes undergo affinity maturation and class switch recombination in germinal centers, which generate memory B cells and long-lived plasmablasts endowed with the capacity to populate specific bone marrow (BM) niches, where resident long lived PCs yield long-lasting serological memory of the pathogen [34, 35].

PC differentiation involves a complex genetic reprogramming network aimed to silence B cell identity and to acquire the distinctive Ig-secretory phenotype, entailing expression of the transcription regulators Blimp-1, IRF4 and XBP1 [36]. Encoded by the *Prdm1* gene, Blimp-1 represses the specific B cell identity transcription factors Pax5 and Bcl-6 [37]. Recent work established that while IRF4 expression is necessary for PC differentiation, Blimp-1 is specifically required for antibody production, but dispensable once ASCs are formed [38]. Moreover, comprehensive gene target assessment revealed that Blimp-1 is not only a repressor of B cell identity genes involved in antigen presentation and class switch recombination (including *Aicda*, which encodes the cytidine deaminase AID), but also a transcriptional activator. Indeed, Blimp-1 promotes Ig transcription and the conversion from the membrane-bound to the secreted form of the Ig heavy chain [39]. Moreover, Blimp-1 induces XBP1, a key transcription factor of the unfolded protein response (UPR) integral to PC differentiation [40, 41].

We have recently established a pivotal role of autophagy in PC ontogeny. Having found concertedly increased expression of autophagy related genes in differentiating PCs *ex vivo*, and remarkable autophagic activity in both short- and long-lived PCs *in vivo*, we then investigated PC ontogenesis in mice in which autophagy is selectively ablated in B cells (*Atg5*f/f CD19-Cre mice) [28]. Attesting to a developmental key role for autophagy in PC differentiation, *Atg5*f/f CD19-Cre mice showed reduced IgM and IgG responses following both T-independent and T-dependent immunizations. Notably, although long-lived PCs were normally represented in the BM of *Atg5*f/f CD19-Cre mice, genomic characterization found no recombined floxed Atg5 in long-lived PCs, revealing efficient Darwinian selection for autophagy-proficient PCs, which demonstrated the absolute requirement for autophagy in BM PCs. Moreover, *Atg5*f/f CD19-Cre mice had a profound defect of antigen-specific long-lived PCs. Thus, autophagy is essential for PC maintenance in the BM and serological memory [28]. An independent confirmation came from a recent work that identified and characterized human BM PCs from a gene expression and functional standpoint. Here, the CD19^−^ CD38^hi^ CD138^+^ population defined a subset of very long-lived PCs that showed abundant autophagy and distinctive high expression of autophagic genes [42].

**Fig. 1:**
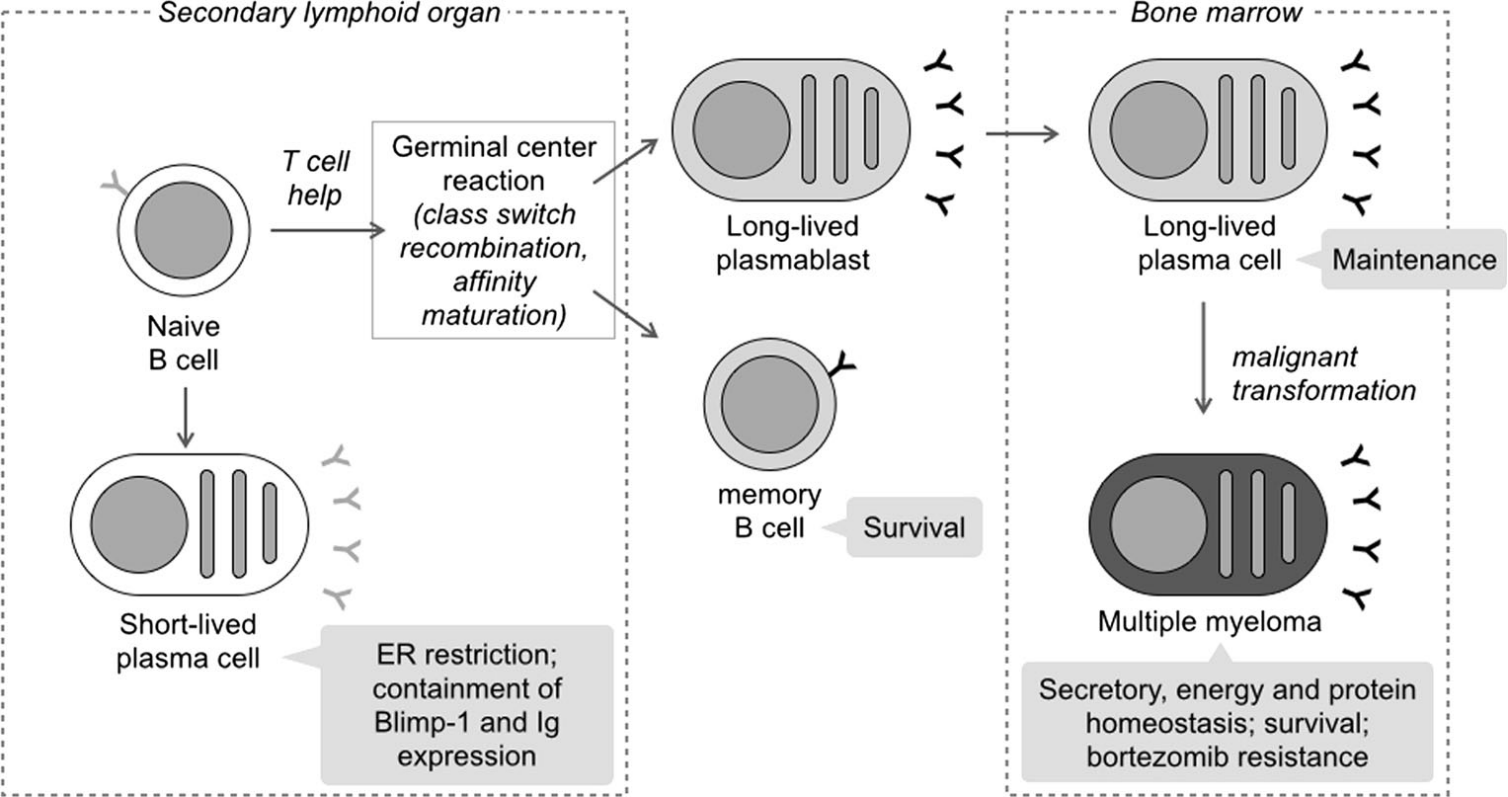
Functions of autophagy in normal and malignant plasma cells. In differentiating plasma cells, autophagy restricts the ER and moderates Blimp-1 expression, containing Ig production to sustainable levels, ultimately supporting antibody responses [28]. B cell autophagy is dispensable for the germinal center reaction [28], while the maintenance of memory B cells and bone marrow long-lived plasma cells depend on autophagy [28, 42, 44]. In multiple myeloma, the malignant counterpart of memory plasma cells, autophagy is required for secretory and energetic homeostasis and survival, and confers specific resistance to the first-in-class proteasome inhibitor, bortezomib [56]. The functions of autophagy hitherto recognized across plasma cell ontogenesis are summarized in *grey boxes*

To understand why PCs require autophagy, we devised an unbiased comparative proteomic analysis of wild-type and *Atg5*^−/−^ differentiating PCs by stable isotope labeling by amino acids in cell culture (SILAC). The proteome of developing PCs could be entirely labeled in as little as 3 days, not only enabling this approach, but also demonstrating the unique degree of proteome plasticity associated with this cellular metamorphosis. Proteome analysis revealed more abundant ER in *Atg5*^−/−^ PCs, further confirmed by qualitative and quantitative electron microscopy techniques, disclosing selective ER-phagy in differentiating PCs. Autophagy-deficient PCs also showed higher expression of the master regulators Blimp-1 and XBP1, and higher Ig expression, manufacturing and secretion [28]. As a result, *Atg5*^−/−^ PCs ran short of ATP and died prematurely, explaining the requirement of autophagy for normal antibody responses *in vivo* [28]. This study has several implications. First, it discloses a new autophagy-dependent mechanism regulating PC differentiation. Puzzlingly, autophagy was shown not to mediate the disposal of Ig byproducts. Hence, a direct link between ER size and enhanced XBP1 signaling is to be excluded, and the mechanism whereby autophagy contains Blimp-1 expression remains elusive. Second, this work unveils a previously unsuspected level of plasticity in PC biology and antibody responses, whose functional significance in immune pathophysiology awaits elucidation. The implication of autophagy in human primary antibody deficiencies will shed light on this issue [43]. Of note, further expanding its significance across humoral immunity, autophagy recently proved essential also for the survival of memory B cells [44]. The functions of autophagy hitherto recognized in PC ontogeny are synoptically summarized in Fig. 1.

## Autophagy in Cancer

The role of autophagy in cancer is complex and multifaceted. Autophagy is primarily a homeostatic process that recycles damaged proteins and organelles, maintaining cellular homeostasis, thereby preventing the accumulation of ROS, inflammation, and oncogenic mutations [45]. In keeping with its broad cytoprotective role, genetic defects of autophagy in mice have been associated with higher susceptibility to metabolic stress, genomic damage, and tumorigenesis, establishing the notion that autophagy is oncosuppressive in normal cells [46]. Monoallelic deletions of the gene encoding the essential autophagic player beclin1 have been detected in 40 to 70 % of human breast, prostate and ovarian cancers; however, the genomic position of beclin1 close to the established tumor suppressor BRCA1 disputes the oncogenic mechanism of such mutations [47]. In absence of autophagy, the inability to clear p62^+^ aggregates has been shown to increase ROS levels, DNA damage and cell death [48, 49]. Moreover, accumulation of p62 has been proposed to be tumorigenic itself through altered NF-kB and Nrf2 activity [50, 51].

On the other hand, autophagy deficiency in mouse liver leads to the development of benign hepatomas, suggesting that autophagy may be protective from neoplastic transformation, but is also involved in the acquisition of malignant features, promoting cancer growth, survival and metastatization [52]. This is in line with the general notion that transformed cells experience more stress due to deregulated growth, hypoxia, nutrient deprivation, and oxidative insults, and hence rely more on adaptive strategies than normal cells. Autophagy may thus be essential for cancer cells to resist metabolic and environmental stress. Indeed, many cancer types show higher autophagic activity than normal counterparts. Moreover, certain tumors may be particularly dependent on autophagy for mitochondrial homeostasis due to peculiar metabolic features, such as impaired acetyl-CoA biosynthesis in RAS-driven cancers. Of therapeutic interest, autophagy is often upregulated by chemotherapy, offering putative targets to overcome primary and secondary drug resistance [45].

## Autophagy in Multiple Myeloma

Multiple myeloma (MM), the malignant counterpart of BM long-lived PCs, is a frequent and incurable tumor accounting for ~2 % of all cancer deaths. MM biology is a unique model to study the role of autophagy in cancer. Intense Ig production is associated with high workload for the UPS; as a result, MM is exquisitely vulnerable to proteasome inhibitors (PIs), but resistance inevitably ensues [53]. Autophagy has been shown to cooperate with the UPS in animal and cellular models of proteotoxicity [54, 55], offering an attractive target to overcome PI resistance. This rationale, together with the essential role of autophagy identified in normal PCs [28], encouraged us to investigate the constitutive function and relevance of autophagy in MM. We found that myeloma cells display remarkable basal autophagic activity as compared to other tumors, including B lymphomas, and that autophagy is necessary for MM cell survival [56]. Wide-scope and hypothesis-driven approaches revealed two main autophagic tasks in myeloma cells: containment of the secretory apparatus, as observed in normal PCs (*vide supra*), and tight cooperation with the UPS for the clearance of ubiquitinated proteins through a p62-dependent autophagic reserve [56] (Fig. 1). Notably, in myeloma cells, autophagy participates in Ub-protein clearance in basal conditions (i.e. in absence of PIs). Despite this close collaboration, combined inhibition of autophagy and the UPS in preclinical studies yielded discrepant results, ranging from synergy to antagonism [56–58]. Adding to complexity, deregulated autophagy has been shown to promote MM cell death, through a non-apoptotic caspase 10-dependent mechanism [59]. Such complexity is likely accounted for by the multiple, integrated functions controlled by autophagy. Hence, dissecting tissue- and cancer-specific tasks, substrates and molecular mechanisms of autophagy is needed to design new targeted therapeutic strategies.

Our study identified p62 as a novel, specific anti-myeloma target [56]. We found that the genetic ablation of p62 induced massive and rapid death of myeloma cells, but not B lymphomas. Moreover, p62 yielded specific PI resistance by mediating a twofold plastic adaptive response to proteasome stress. First, PIs rapidly induced *de novo* expression of p62, but not other autophagy receptors. Second, PIs dramatically modified the interactome of p62, inducing its rapid nucleation onto detergent-insoluble aggregates, at the expense of numerous signaling interactors, as revealed by fluorescent microscopy and quantitative proteomics [56]. Hence, under PIs, MM cells intensify p62-dependent autophagic degradation so as to compensate for proteasome insufficiency, but do so by neglecting other cytoprotective functions. Thus, understanding which integrative role of p62 is crucial for myeloma viability holds promise to disclose new therapeutic targets. Of interest, we found that p62-silenced myeloma cells rapidly lose intracellular ATP prior to cell death, in keeping with a role of p62 in mitochondrial homeostasis [56]. One possible mechanism is the maintenance of mitochondrial health via mitophagy, whose mechanisms are still debated. A paradigmatic pathway involves the proteins PINK and Parkin. The former is a short-lived kinase that localizes to mitochondria. Upon mitochondrial depolarization, PINK is stabilized and recruits the cytosolic ubiquitin-ligase Parkin to ubiquitinate proteins on the outer mitochondrial membrane, marking damaged mitochondria for elimination. The autophagic receptors involved are under investigation, with a recent work implicating NDP52 and optineurin, and not p62, in PINK-mediated mitophagy [60]. In fact, the homeostatic and integrative functions linking autophagy and mitochondria may extend beyond mitophagy, as witnessed by the intense relationship between p62 and the mitochondrial network [56].

## Conclusions and Perspectives

Autophagy is a tightly regulated intracellular recycling process underlying organelle quality control, proteome plasticity, cell differentiation and stress responses, with essential roles in health and disease. Selective target recognition by specific adapter proteins enables autophagy to orchestrate highly specialized tissue-specific tasks, including functions in innate and adaptive immunity. The recently identified negative control exerted by autophagy on the Blimp-1-dependent differentiation program shapes PCs for sustainable antibody production. This control discloses a previously unsuspected level of plasticity of PC biology, whose mechanisms and pathophysiological significance in humoral immunity warrant investigation. On the malignant side, in MM cells autophagy collaborates closely with the UPS for protein homeostasis, providing a framework to overcome PI resistance. Moreover, the exquisite dependence of malignant PCs on the autophagy adapter and hub signaling integrator p62 recommends that autophagy-regulated complex integrative functions be dissected to design novel targeted therapies against myeloma.

## Abbreviations

ASC: antibody secreting cells
BM: bone marrow
ER: endoplasmic reticulum
Ig: immunoglobulin
LAP: LC3-associated phagocytosis
MM: multiple myeloma
PC: plasma cell
ROS: reactive oxygen species
SILAC: stable isotope labeling by amino acids in cell culture
TCR: T cell receptor
Ub: ubiquitin
UPR: unfolded protein response
UPS: ubiquitin-proteasome system
